# Effect of a Multicomponent mHealth Intervention on the Composition of Diet in a Population with Overweight and Obesity—Randomized Clinical Trial EVIDENT 3

**DOI:** 10.3390/nu14020270

**Published:** 2022-01-09

**Authors:** Cristina Lugones-Sánchez, José I. Recio-Rodríguez, Marta Menéndez-Suárez, Alicia Saz-Lara, José I. Ramirez-Manent, María A. Sánchez-Calavera, Leticia Gómez-Sánchez, Emiliano Rodríguez-Sánchez, Luis García-Ortiz, Evident 3 Investigators Group

**Affiliations:** 1 Primary Care Research Unit of Salamanca (APISAL), Institute of Biomedical Research of Salamanca (IBSAL), Health Service of Castilla y León (SACyL), 37005 Salamanca, Spain; donrecio@gmail.com (J.I.R.-R.); leticiagmzsnchz@gmail.com (L.G.-S.); emiliano@usal.es (E.R.-S.); lgarciao@usal.es (L.G.-O.); 2 Department of Nursing and Physiotherapy, University of Salamanca, 37007 Salamanca, Spain; 3 University Clinical Hospital of Valladolid, Health Service of Castilla y León (SACyL), 47003 Valladolid, Spain; martamenendezsuarez@gmail.com; 4 Health and Social Research Center, University of Castilla-La Mancha, 16071 Cuenca, Spain; Alicia.delSaz@uclm.es; 5 Calvià Primary Care Center, Balearic Islands Health Research Institute (IDIBSA), Health Service of Balearic Islands, 07180 Calvià, Spain; iramirez@ibsalut.caib.es; 6 Department of Medicine, University of the Balearic Islands, 07122 Palma, Spain; 7 Las Fuentes Norte Health Center, Aragonese Group of Primary Care Research (GAIAP), Aragon Health Research Institute (IISA), Aragon Health Service, 50002 Zaragoza, Spain; mascalavera62@hotmail.com; 8 Department of Internal Medicine, Psychiatry and Dermatology, University of Zaragoza, 50009 Zaragoza, Spain; 9 Department of Medicine, University of Salamanca, 37007 Salamanca, Spain; 10 Department of Biomedical and Diagnostic Sciences, University of Salamanca, 37007 Salamanca, Spain; 11 EVIDENT Research Group, RICAPPS: Chronicity, Primary Care and Health Promotion Research Network, 37005 Salamanca, Spain; apisal2020@gmail.com

**Keywords:** mobile app, telemedicine, obesity, healthy diet, diet records

## Abstract

A balanced diet can help in the prevention of chronic diseases. The aim of this study was to evaluate the effect of an mHealth intervention on the distribution of macronutrients and the intake of food groups. A total of 650 participants were included in this multi-center, clinical, randomized, controlled trial (Evident 3 study). All participants were given brief advice about diet and exercise. The intervention group received, in addition, an app (Evident 3) for the self-recording of their diet and an activity tracker wristband for 3 months. Follow-up visits were performed at 3 and 12 months to collect the diet composition using the Food Frequency Questionnaire. There were decreases in the intake of total calories, fat, protein and carbohydrates in both groups throughout the study, without significant differences between them. The intervention group reduced the intake of cholesterol (−30.8; 95% CI −59.9, −1.7) and full-fat dairies (−23.3; 95% CI −42.8, −3.8) and increased the intake of wholemeal bread (3.3; 95% CI −6.7, 13.3) and whole-grain cereals (3.4; 95% CI −6.8, 13.7) with respect to the control group. No differences were found in the rest of the nutritional parameters. The brief advice is useful to promote a healthier diet, and the app can be a support tool to obtain changes in relevant foods, such as integral foods, and the intake of cholesterol. Trial registration: ClinicalTrials.gov with identifier NCT03175614.

## 1. Introduction

Changes towards less healthy lifestyles, such as unbalanced diets and the consumption of ultra-processed products, along with physical inactivity, have caused an increase in the prevalence of obesity in the population [1,2]. Moreover, other studies have reported that poor-quality diets, that is, with little nutritional value [3] and a low score in diet quality indices [4], are associated with cardiovascular risk factors [5], leading to an increase in the prevalence of cardiometabolic diseases [6,7,8] and in mortality by chronic diseases [9,10]. Similarly, an inadequate intake of whole-grain cereals, fruit and sodium has been identified as a risk factor, relating to 50% of deaths and 66% of disability-adjusted life years [11]. Therefore, it seems necessary to develop interventions that promote a more balanced diet at every age range, even if these do not result in a short-term weight loss [12].

With the aim of promoting healthier lifestyles and reducing obesity, several studies have evaluated the effectiveness of nutritional interventions based on restricting the intake of calories, carbohydrates and fats in overweight populations, obtaining some beneficial results, such as weight loss [13,14], and improving other health variables [15,16]. However, the effect of this type of interventions is not always maintained in the long term [17,18], thus new strategies are required to prolong the effect. 

In this sense, new technologies can play an important role, offering more personalized solutions adapted to the needs of the individual. Moreover, the use of mHealth can provide greater flexibility for their use, facilitating the long-term maintenance of nutritional interventions. In recent years, different studies have evaluated several digital tools for the self-monitoring of diet as a support for the treatment of obesity. Some of these tools have proved to be effective in body weight loss and in the reduction in energy consumption with respect to traditional interventions [19,20]. Mobile applications with other approaches to achieve a change of habits, such as those that include a personalized food planning [21] and those which provide nutritional information after scanning the bar code of food products [22], have also obtained promising results. All this indicates that this type of digital interventions can help to modify eating behaviors, and, consequently, improve different health parameters [23,24].

However, most of the studies that have included this type of tools have mainly evaluated weight loss, the increase in physical activity or the reduction in dietary energy consumption, thus there is still little evidence of the effect of these interventions on diet composition [25]. This information can be important, since obtaining changes in the amount of healthy foods consumed, such as fruit and vegetables, can be a good starting point to attain a more effective and long-lasting change of lifestyle, resulting in the long-term improvement of other health parameters [17], which could prevent the development of chronic diseases [26].

Therefore, the aim of this study was to evaluate the effect of a multicomponent intervention, with a mobile app for diet recording and an activity tracker wristband, on the diet composition, the distribution of macronutrients and the intake of food groups in a Spanish overweight and obese population. 

## 2. Materials and Methods

### 2.1. Study Design

The EVIDENT 3 study is a multicenter, randomized, controlled clinical trial with two parallel groups and a 12-month follow-up. The study was conducted in five healthcare centers of different Spanish cities (Salamanca, Valladolid, Zaragoza, Cuenca and Palma de Mallorca) belonging to the Spanish Research Network for Preventive Activities and Health Promotion in Primary Care (REDIAPP). Each participant completed a baseline visit and two follow-up visits (at 3 and 12 months) from randomization. The visits of the EVIDENT 3 study were carried out between June 2017 and June 2020. The study was previously registered as a clinical trial (ClinicalTrials.gov identifier: NCT03175614).

### 2.2. Participants

The sample was selected by consecutive sampling among the individuals who visited the primary care professionals of the participating centers. The selection criteria were: 18–65 years of age, a body mass index (BMI) of 27.5–40, being classified as a sedentary person (performing 20 min of vigorous physical activity less than 3 times per week; 30 min of moderate physical activity less than 5 times per week; or any combination of these intensities less than 5 times per week [27]) and signing the informed consent. The study excluded those individuals with previous cardiovascular diseases, type 1 or 2 diabetes mellitus, people with a BMI over 40 kg/m^2^, and those who were on a diet monitored by a healthcare professional. The detailed list of inclusion and exclusion criteria can be checked in the protocol [28]. 

### 2.3. Screening and Randomization

**Figure 1:**
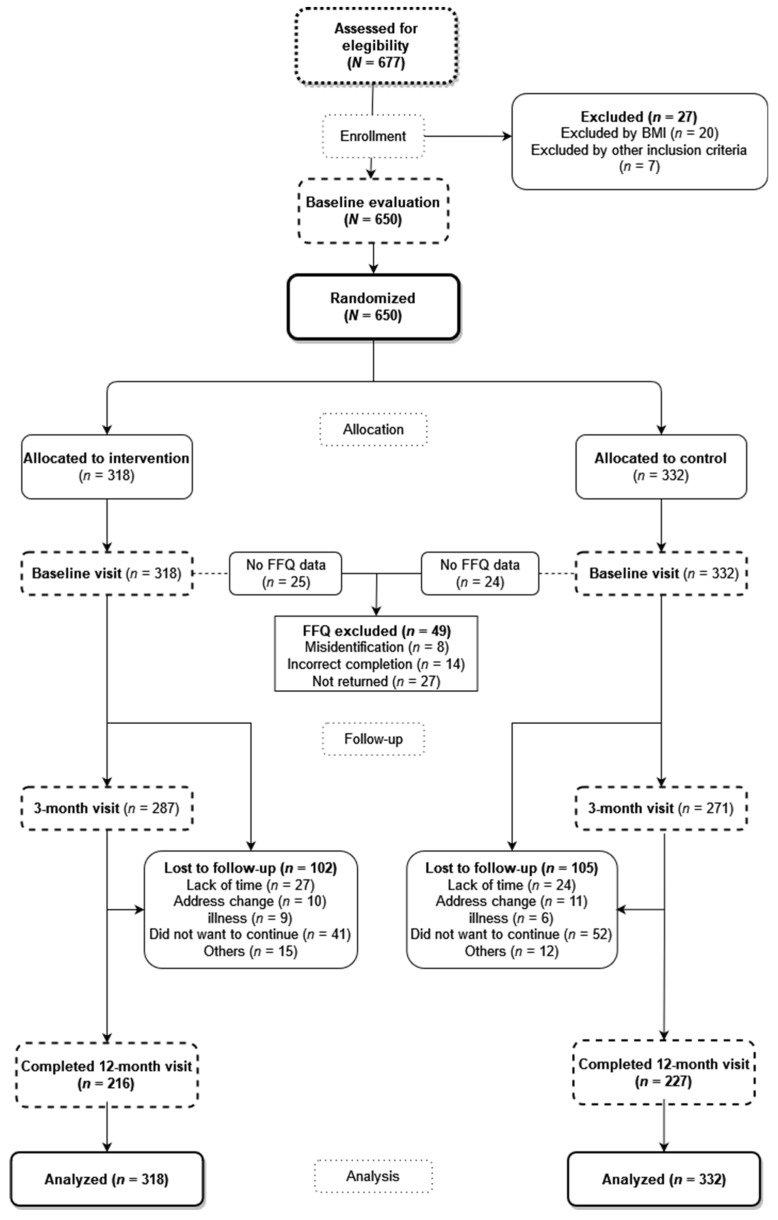
Flowchart of the study. FFQ: Food Frequency Questionnaire.

A total of 677 individuals were evaluated for inclusion, of whom 27 candidates were excluded (20 did not meet the BMI criterion, and the other 7 were excluded for other reasons) (Figure 1). The sample included in the study consisted of a total of 650 individuals randomized into two groups with a ratio of 1:1. The control group (CG), who only received a brief advice, was composed of 332 individuals, and the intervention group (IG), who received, in addition to the brief advice, the app and the activity tracker wristband for 3 months, consisted of 318 individuals. The randomization sequence was generated with a software (Epidat 4.2, Xunta de Galicia, Spain) by an independent researcher, who was blinded until she performed the randomization of the participants to the groups. 

To minimize the possible contamination between the groups, the researcher who performed the intervention was different from the one who conducted the evaluation visits. The researcher who performed the data analysis was also blinded. Due to the nature of the intervention, the subjects could not be blinded to it.

### 2.4. Intervention

#### 2.4.1. Intervention Common for Both Groups

A nurse of each participating healthcare center, who were previously trained for the study and unconnected to the other activities of the study, gave the standardized individual brief advice about healthy lifestyles before randomization to the study groups. The advice regarding diet was based on the plate method [29] to attain a more balanced diet, where the plate is divided into 4 parts: half of the plate for salad or vegetables, a quarter for carbohydrates and the other quarter for proteins (preferably white meats). Moreover, it is recommended to add a medium-sized piece of fruit and a low-fat dairy product, which can be consumed as a dessert. The nurses explained the benefits of performing physical activity and the international recommendations about weekly physical activity, recommending to perform at least 30 min of moderate exercise (cycling, doubles tennis, etc.) 5 days per week or 20 min of vigorous activity (swimming, running, etc.) 3 days per week.

#### 2.4.2. Specific Intervention 

After completing the baseline visit and giving the standardized advice to the participants, another visit was performed 7 days after the baseline visit, where all the participants were asked to return the accelerometer and the questionnaires completed by them. Additionally, the participants of the IG received a Smartphone (Samsung Galaxy, Samsung, Suwon, Korea) with the mobile app and an activity tracker wristband (Xiaomi Miband 2, Xiaomi, Beijing, China), to be used throughout the 3 months of the intervention, training them in the use of these tools in a 15-min session on the day they were given these devices. Both the app and the rest of the materials used were provided in Spanish.

**Figure 2:**
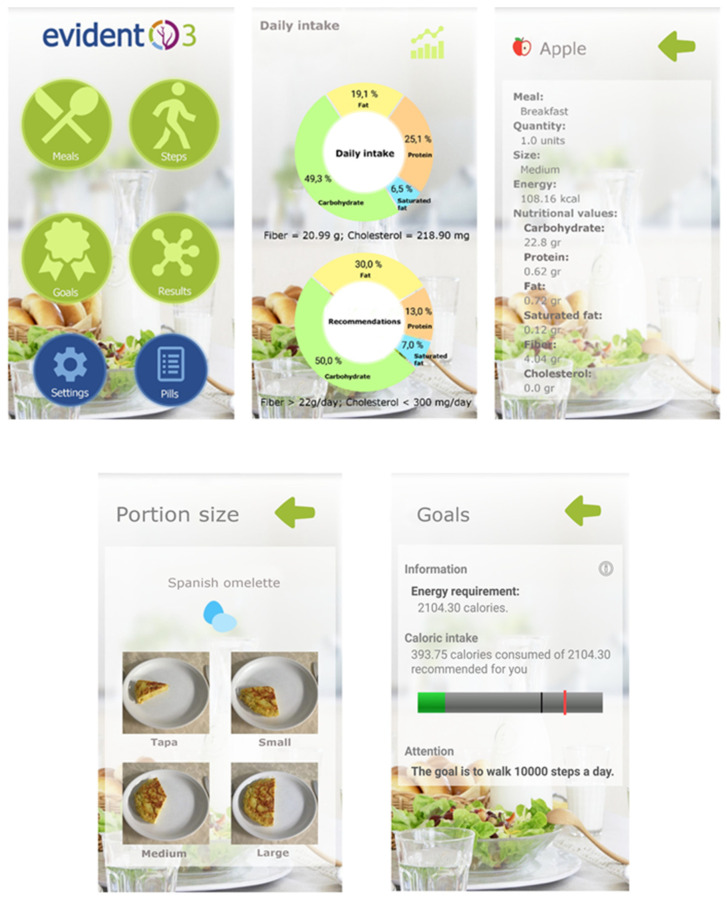
Screenshots of the Evident 3 app.

The application was set up with the participant’s data (sex, age, weight and height) to set the goals to be attained. The application was designed to enable the self-recording of the daily diet and the automatic gathering of the physical activity recorded by the activity tracker wristband. The participants recorded their daily diet, by selecting, within each meal (breakfast, brunch, lunch, tea and dinner), the foods or dishes consumed among the options offered by the app, indicating the portion size from among the images displayed (tapa, small, medium and large). The nutritional composition of the foods and dishes present in the app were gathered from the Spanish Food Composition Database (BEDCA) [30], which was developed by RedBEDCA and the Spanish Agency of Food and Nutrition (AESAN). Once the user introduces all the information, the app integrates all the data to create personalized recommendations and goals for energy intake, displaying the limits as a black and red line (Figure 2). The upper limit (the red line) was the result of adding the basal metabolic rate, diet-induced thermogenesis and estimated energy expenditure for sedentary activities, while the lower limit (the black line) was the 85% of those calculated calories. The participants could check these recommendations and the information about the daily energy consumption and the distribution of macronutrients (carbohydrates, proteins, and saturated and unsaturated fats) within the app (Figure 2). The application includes the use of effective behavioral strategies to achieve changes in habits, such as self-recording [31], goal setting and positive reinforcement [32]. A multidisciplinary team of doctors, nurses, dietitians and psychologists has collaborated in the development of the app. After the 3 months of intervention, all the devices were collected. All the information generated by the application was analyzed and integrated into the databases of the study.

### 2.5. Measurements

#### 2.5.1. Dietary Intake

The dietary intake was recorded through the Food Frequency Questionnaire (FFQ), which is self-administered and semi-quantitative, consists of 137 items about eating and has been validated for use in the Spanish population [33]. The participants were requested to complete it with the frequency at which they had consumed, on average, each of the foods of the questionnaire during the last year, choosing one of the nine possible frequencies: never or almost never, 1–3 times per month, once per week, 2–4 times per week, 5–6 times per week, once per day, 2–3 times per day, 4–5 times per day, 6 times per day or more. The diet of the participants was evaluated with this questionnaire both in the baseline visit and in the follow-up visits (at 3 and 12 months). This questionnaire was handed to the participants after each visit, and they were requested to complete it at home and return it 7 days later, along with the devices, for subsequent analysis. The obtained information allowed for estimating the energy consumption, the distribution of macro and micronutrients and the daily intake of each macronutrient and food group in grams per day. 

#### 2.5.2. Other Measurements

The detailed description of the variables of the study and how they were gathered can be checked in the published study protocol [28]. 

Sociodemographic data: information was gathered about age, sex, marital status, employment situation and education level. 

Motivation for change: in the baseline visit, the participants completed a self-developed questionnaire to evaluate the motivation and self-efficacy for change, following the transtheoretical model of Prochaska and DiClemente for health behavior change and weight management [34,35]. The questionnaire consisted of 6 Likert-scale items with 5 possible answers, and the participants were classified into three groups according to the obtained score: “not ready” (pre-contemplation and contemplation stages, ≤15 points), “ready” (preparation stage, 16–24 points) and “at the right moment” (action stage, ≥25 points).

Laboratory variable assessment: blood and urine samples were gathered early in the morning, with the patient fasting for over 12 h, following the general recommendations for the determination of the lipid profile, plasma glucose and insulin in the laboratory of the reference hospital. 

Peripheral blood pressure: Three measurements of systolic blood pressure (SBP) and diastolic blood pressure (DBP) were recorded using the average of the last two measures with a validated Omron M10-IT sphygmomanometer (Omron Healthcare, Kyoto, Japan). The measurements were performed in both arms, with the participant sitting, after at least 5 min of rest, with a muff of adequate size and following the recommendations of the European Society of Hypertension [36]. 

Smoking habit: This was evaluated through a questionnaire with 4 standard questions adapted to the MONICA study of the WHO [37]. The participants of the study were classified as current smokers, former smokers (over one year without smoking) or non-smokers.

### 2.6. Ethics Approval and Consent to Participate

The study was approved by the Drug Research Ethics Committee of the Health Area of Salamanca (Approval Code: 2016-04-P1600170; Approval Date: 25 April 2016) (“CEIm del Área de Salud de Salamanca”) in April 2016 as coordinating team. It was also approved by the Ethics Committee of the rest of the collaborating centers: Western Valladolid, Aragón, Castile-La Mancha and Palma de Mallorca. The participants received the information of the study and signed the informed consent before being included in the study, in compliance with the guidelines of the Declaration of Helsinki [38].

### 2.7. Stadistical Analysis

The sample size was estimated for the main variables of the study. The recruitment of 650 individuals, with 318 participants in the intervention group and 332 in the control group, was considered sufficient to detect a statistically significant difference of 10 g of total intake of fat between the two groups, with alfa and beta risk levels of 0.05 and 0.20, respectively, in a bilateral test, a standard deviation of 35 g and a correlation between measurements of 0.5, estimating a follow-up loss rate of 10% [39].

The results are expressed as mean ± standard deviation for the quantitative variables or through the number and frequency distribution for the categorical variables. The results were analyzed by intention-to-treat, including all the patients randomized in the group to which they were assigned, regardless of their adherence to, abandonment of or deviation from the protocol, as well as everything that occurred after the randomization [40]. No imputation was performed for the missing data of the FFQ. Student’s *t*-test was used to compare the means between the two groups, and the paired t-test was employed to evaluate the changes within the same group. 

To compare the changes in the composition of the diet between the groups along time (baseline, 3 months and 12 months), a variance analysis was conducted for repeated measures. The presence or absence of sphericity was taken into account, and the Greenhouse-Geisser correction was conducted when necessary. Contrast hypotheses were established using α = 0.05. The data were analyzed with IBM SPSS Statistics for Windows v.26.0 (IBM Corp, Armonk, NY, USA).

## 3. Results

### 3.1. Baseline Characteristics

Of all the individuals included in the study, 67.3% (*n* = 214) and 69.6% (*n* = 231) were women in IG and CG, respectively, with an average age of 47.7 ± 10.1 and 48.9 ± 9.2 years, respectively, and with no differences in the demographic or baseline clinical characteristics between the two groups (Table 1). Of the 650 individuals included in the trial, 102 and 105 in IG and CG, respectively, did not perform the two follow-up visits due to different reasons (see Figure 1). Therefore, a total of 443 individuals (216 in IG and 227 in CG) completed the follow-up evaluation at 12 months. 

The comparison between the baseline characteristics of the 207 individuals who abandoned the study and those who completed it is shown in Table A1. No differences were found between the groups in the analysis of the stage of dietary habit change in the baseline visit, with most of the sample being ready (IG: 52.5%; CG: 52.1%) or at the right moment for change (IG: 42.8%; CG: 44.3%).

In the baseline visit, the FFQ questionnaire was completed and returned by 293 (92.1%) participants of IG and 308 (92.7%) of CG, excluding 49 participants for the causes mentioned in Figure 1. In the visit at 3 months, 236 and 232 questionnaires were collected in IG and CG, respectively, and, in the visit at 12 months, 190 and 197 questionnaires were collected in IG and CG, respectively.

### 3.2. Changes in Macronutrients Intake

**Figure 3:**
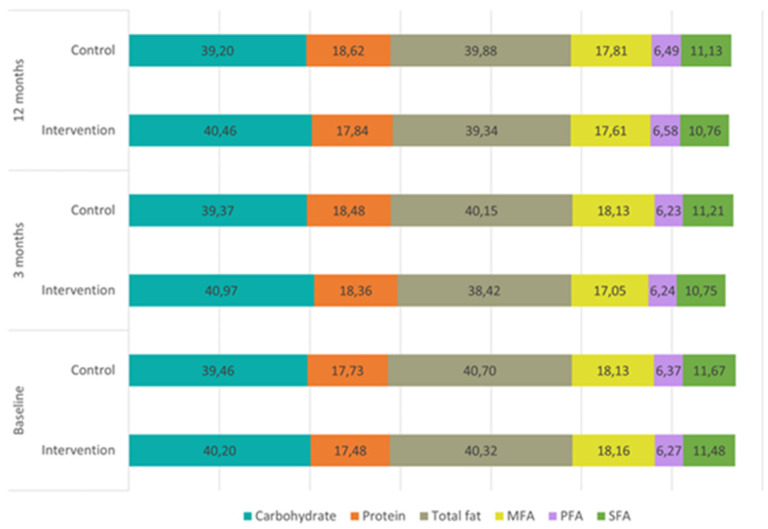
Dietary composition by energy percentage of the main nutrients per visit for each study group. SFA: Saturated Fatty Acids; PFA: Polyunsaturated Fatty Acids; MFA: Monounsaturated Fatty Acids.

The dietary composition of the two groups throughout the study is represented in Figure 3, showing no differences between them. In the visit at 3 months, IG reduced the intake of carbohydrates (−18.3; 95% CI −29.4, −7.3), proteins (−3.3; 95% CI −6.3, −0.3), total fat (−13.4; 95% CI −17.4, −9.4), and all fatty acids and cholesterol (Table 2), and increased the dietary fiber (1.1; 95% CI 0.1, 2.0). In the visit at 12 months, this decrease in macronutrients was maintained, whereas the increase in the intake of fiber was not. 

On the other hand, CG showed similar results at 12 months, with reductions in the three main macronutrients: carbohydrates (−27.2; 95% CI −38.5, −15.9), proteins (−3.9; 95% CI −7.4, −0.5) and total fat (−10.4; 95% CI −15.2, −5.7). However, this group did not show changes in the intake of fiber in the follow-up visits. 

**Figure 4:**
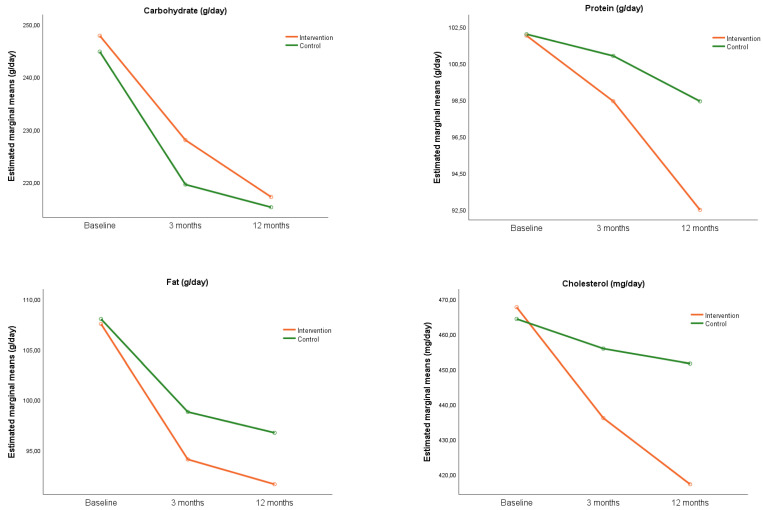
Evolution of the intake of macronutrients throughout the study.

Figure 4 shows the evolution of the intake of the main macronutrients and cholesterol for each group during the study. The analysis of the effect of the intervention on macronutrients intake (Table 3), found no differences between groups at 3 and 12 months except for cholesterol intake, where a decrease in IG was found compared to CG (−30.8; 95% CI: −59.9, −1.7) at 12 months. 

### 3.3. Changes in Daily Intake of Food Groups

Table 4 shows the changes obtained in the visits of the study for both groups. In the visit at 3 months, IG reduced the intake of olive oil, full-fat dairy products, biscuits, sweets, industrial pastries, sodium and sugar, and increased the intake of wholemeal bread. On the other hand, CG reduced the intake of biscuits, sweets, industrial pastries, sodium and sugar, and increased the intake of vegetables, fruit, legumes and white meat. Therefore, improvements were obtained in the diet of both groups.

In the visit at 12 months, both groups increased the intake of vegetables (IG: 24.8; 95% CI 7.9, 41.7 and CG: 25.4; 95% CI 8.7, 42.1) and fruit (IG: 34.3; 95% CI 12.4, 56.1 and CG: 37.9; 95% CI 16.0, 59.8). The two groups also reduced the intake of dairy products, red meat, biscuits, sweets, industrial pastries, sodium and sugar. In addition to these changes, IG increased the dairy grams of nuts (2.4; 95% CI 0.01, 4.8) and wholemeal bread (7.2; 95% CI 0.3, 14.0), and reduced the intake of sugary drinks (−12.1; 95% CI −20.6, −3.6). Table 5 shows the effect of the intervention on the different food groups, obtaining an increase in IG with respect to CG for wholemeal bread (14.7; 95% CI 6.4, 23.0) and whole-grain cereals (15.7; 95% CI 7.3, 24.1) at 3 months, and for full-fat dairy products (−23.3; 95% CI −42.8, −3.8), wholemeal bread (3.3; 95% CI −6.7, 13.3) and whole-grain cereals (3.4; 95% CI −6.8, 13.7) at 12 months.

## 4. Discussion

The intervention of the EVIDENT 3 study, consisting of a mobile application for the recording of the diet and an activity tracker wristband, in combination with a brief advice, obtained relevant changes in the habitual dietary composition, measured through the self-administered FFQ questionnaire. Specifically, there was a decrease in the intake of cholesterol and full-fat dairy products, and an increase in the intake of wholemeal bread and whole-grain cereals in IG with respect to CG at 12 months. Moreover, both groups decreased the total intake of calories and essential nutrients, and increased the consumption of vegetables and fruit. Additionally, the reduction in the grams per day of confectionery, industrial pastries, sodium and sugar in both groups at the follow-up visits should be noted, although no differences were found between groups.

The use of digital tools, such as apps containing self-recording strategies, have proved to be beneficial for the change towards healthier behaviors [41], allowing the user to obtain greater knowledge about his/her diet. A recent systematic review has shown that the applications that include a self-record of diet have great potential to improve the intake of fruit and vegetables, with consequent benefits for health [42]. Moreover, they seem to have greater acceptance among users than the traditional self-recording methods [41,43]. In fact, these strategies have been used in previous studies for the modification of the quality of life, increasing the intake of healthier food groups, such as the VegEze app, which promoted the intake of vegetables and obtained an increase in a daily half ration of vegetables [44], and the Vegethon app [45], which obtained an increase in a ration of vegetables measured with the 24-h diet recorder. Despite the fact that the intervention of our study did not have an effect on the intake of fruit and vegetables, it is worth highlighting that neither the recommendation nor the self-record are exclusively focused on these food groups, which is the case of the above-mentioned apps.

The effectiveness of this type of intervention on diet modification was also evaluated with the EVIDENT 2 app, obtaining a decrease in the intake of red meats and a change in the intake of macronutrients [39]. However, this was evaluated in the general population, with a lower proportion of people with obesity, thus the motivation of the participants to adopt healthier habits may have influenced the fact that changes were observed in more food groups.

The results obtained in the EVIDENT 3 study are in line with those obtained with the Mynutricart app [46], which reduced, in IG, the daily ration of refined flour (IG: −1.18, *p* = 0.01) legumes (IG: −0.16, *p* = 0.02) and snacks (IG: −0.78, *p* = 0.03), among others, showing no differences at 8 weeks with respect to CG. Nevertheless, the aim of this app is to facilitate a list of foods for the purchase of healthier options and provide results in the short-term. Therefore, the results of the EVIDENT3 study can provide evidence on the long-term effect of these interventions, reducing the intake of full-fat dairy products and increasing the intake of wholemeal bread and whole-grain cereals at 12 months. On the other hand, no differences were observed in dietary fiber, which could be due to the increase in the intake of fiber recorded in CG from other sources, such as fruit and vegetables. Despite this, an increase in the intake of these food groups allows obtaining a better lipid profile in the medium-long term, decreasing the cardiovascular risk [47,48]. Both wholemeal bread and whole-grain cereals are more nutritional and satiating, and they could also reduce postprandial glucose and increase insulin sensitivity [49,50]; these are also related to a lower risk of gaining weight [51], which can thereby reduce the risk of developing chronic diseases in the long term. 

Similarly, this intervention obtained a decrease in the intake of cholesterol, which is in line with other studies, such as the SMART study [52], which gave a brief advice to the entire sample and a digital self-recording intervention with personalized advices, finding nutritional improvements in the two groups. This intervention obtained, in IG, a decrease in the intake of total calories (IG: −23.4% vs. CG: −14.1%, *p* = 0.03) and saturated fat (IG: −9.3% vs. CG: 3.4%, *p* = 0.04) at 24 months, and in the intake of total fat (IG: −10.4% vs. CG: −4.7%, *p* =0.09) at 6 months, although the latter was not significant. This decrease in the intake of cholesterol could be mainly due to a lower consumption of red meat and full-fat dairy products, since the intake of these was reduced in both groups at 12 months. The reduced intake of cholesterol can improve the levels of plasma cholesterol and reduce the cardiovascular risk in the long term [53]. 

The results show an improvement in the composition of diet in both groups, reducing carbohydrates and fats, with slight differences between the groups due to several factors. The individuals with overweight and obesity who participated in the study were motivated to change, with most of the sample being ready or at the right moment to modify their diet. This may have led CG, who received a brief advice about eating and exercise, to modify their lifestyles towards healthier ones, obtaining similar results at 12 months with respect to IG. It is also worth highlighting that the brief advice given in this study had been previously evaluated [54], proving effective to obtain changes in the main variables of the study, and showing that it may produce differences not in the food groups included in the advice (vegetables, carbohydrates, proteins and fruit) but in those in which the app gave a personalized advice (fat-free dairy products, wholemeal bread and whole-grain cereals). Moreover, there was no additional contact or reinforcement of the advice on healthy habits to the participants in the follow-up visits; therefore, all this could indicate that the brief advice is effective to attain changes in the diet, and that the app could be a support tool that improves the profile of more specific food groups. It is also possible that the intervention time, i.e., 3 months, may have not been enough to generate longer lasting changes in the IG participants; thus, terminating the use of the app caused a progressive loss of the effect of the intervention. In this line, a systematic review has suggested that the effect of eHealth interventions to improve the diet is greater if the intervention time is 4-6 months [55], thus future studies should evaluate the effect of longer interventions on the modification of such variables.

### Limitations

Due to the inclusion of healthy adults with a certain BMI range, the results may not be generalized to other BMI ranges. Despite the fact that the participants were asked not to use any other application or device that could interfere with the study, it cannot be guaranteed that they followed our request. The intervention time (3 months) may have not been enough to identify greater benefits in the composition of the diet. The evaluation of the diet was conducted through a self-informed questionnaire; therefore, there may have been social approval bias, increasing the frequency of healthy foods in the follow-up visits [56]. Moreover, the lack of knowledge of the standard ration of certain foods, such as vegetables (200 g), in the baseline visit may have led to an overestimation of the initial intake, thereby obtaining more accurate information in the follow-up visits after the intervention and the nutritional advice. Future studies should consider the use of more objective measures to obtain more precise results. Lastly, the rate of participants who left the study (31.8%) may have produced biases in the final composition of the sample. However, the randomization of the study group and the fact that similar abandonment rates were obtained in both groups enabled their comparison [57].

## 5. Conclusions

The brief advice given to all participants obtained positive changes both in the macronutrients and in the intake of certain food groups in the sample. Furthermore, the intervention group reduced the intake of cholesterol and full-fat dairy products, while increasing the intake of wholemeal bread and whole-grain cereals. Therefore, the standardized brief advice is useful to promote changes in the diet, and the EVIDENT 3 app can be a support tool to obtain additional changes in some relevant foods, such as integral foods, and in the intake of cholesterol. Further research is necessary to evaluate the effect of this type of intervention on the modification of the diet towards healthier nutritional habits, both in populations with chronic diseases and in general populations, as well as their maintenance in the long-term. 

## Figures and Tables

**Table 1:** Characteristics of study participants at baseline.

		Intervention (*n* = 318)	Control (*n* = 332)	*p*-Value
		Mean ± SD	Mean ± SD	
Age, years		47.7 ± 10.1	48.9 ± 9.2	0.130
Sex, *n* (%)	Men	104 (32.7)	101(30.4)	0.555
	Women	214 (67.3)	231 (69.6)	
Smoking status, *n* (%)	Non smoker	124 (39.0)	139 (41.9)	0.560
	Smoker	68 (21.4)	75 (22.6)	
	Former smoker	126 (39.6)	118 (35.5)	
Marital status, *n* (%)	Single	60 (18.9)	74 (22.3)	0.736
	Married	222 (69.8)	222 (66.9)	
	Divorced	31 (9.7)	30 (9.0)	
	Widowed	5 (1.6)	6 (1.8)	
BMI (kg/m^2^)		33.1 ± 3.4	33.0 ± 3.6	0.607
SBP (mmHg)		119 ± 15	120 ± 16	0.402
DBP (mmHg)		79 ± 9	81 ± 10	0.091
Heart rate (lpm)		72 ± 12	74 ± 12	0.061
Total Cholesterol (mg/dL)		198 ± 36	202 ± 40	0.230
HDL Cholesterol (mg/dL)		51 ± 13	52 ± 12	0.557
LDL Cholesterol (mg/dL)		122 ± 31	125 ± 36	0.247
Triglycerides (mg/dL)		131 ± 73	127 ± 63	0.513
Glycaemia (mg/dL)		93 ± 14	95 ± 21	0.190
HbA1c (%)		5.4 ± 0.4	5.5 ± 0.5	0.058
Hypertension, *n* (%)		88 (27.7)	116 (35.0)	0.052
Dyslipidemia, *n* (%)		73 (23.4)	87 (26.5)	0.411
Diabetes Mellitus *n* (%)		5 (1.7)	4 (1.3)	0.748
Motivation, state to change, *n* (%)	Not ready	15 (4.7)	12 (3.6)	0.754
	Ready	167 (52.5)	173 (52.1)	
	Right moment	136 (42.8)	147 (44.3)	

SD: standard deviation; BMI: body mass index; SBP: Systolic blood pressure; DBP: Diastolic blood pressure; HDL: High-density lipoprotein; LDL: Low-density lipoprotein; HbA1c: Hemoglobin A1c.

**Table 2:** Changes in macronutrients and energy intake throughout the study.

	Intervention Group (*n* = 293)			Control Group (*n* = 308)		
	Baseline Mean (SD)	3 Months Mean Diff. (95 CI%)	12 Months Mean Diff. (95 CI%)	Baseline Mean (SD)	3 Months Mean Diff. (95 CI%)	12 Months Mean Diff. (95 CI%)
Carbohydrates (g/day)	242.0 (81.9)	−18.3 (−29.4 to −7.3) **	−31.4 (−43.6 to −19.2) **	235.8 (86.9)	−22.1 (−32.2 to −12.0) **	−27.2 (−38.5 to −15.9) **
Proteins (g/day)	102.4 (26.2)	−3.3 (−6.3 to −0.3) *	−8.8 (−12.4 to −5.1) **	102.0 (24.8)	−2.6 (−5.9 to 0.7)	−3.9 (−7.4 to −0.5) *
Total fat (g/day)	107.6 (35.9)	−13.4 (−17.4 to −9.4) **	−15.2 (−20.3 to −10.0) **	106.8 (35.3)	−9.4 (−13.6 to −5.3) **	−10.4 (−15.2 to −5.7) **
Monounsaturated fatty acids (g/day)	48.5 (18.2)	−6.6 (−8.7 to −4.5) **	−7.0 (−9.7 to −4.2) **	47.6 (17.4)	−3.9 (−6.0 to −1.8) **	−5.0 (−7.3 to −2.7) **
Polyunsaturated fatty acids (g/day)	16.8 (6.8)	−1.2 (−2.0 to −0.4) **	−1.3 (−2.4 to −0.2) *	16.8 (7.4)	−1.7 (−2.6 to −0.8) **	−1.0 (−2.1 to 0.2)
Saturated fatty acids (g/day)	30.7 (10.6)	−4.5 (−5.6 to −3.3) **	−5.6 (−7.0 to −4.2) **	30.7 (10.7)	−3.3 (−4.5 to −2.0) **	−3.7 (−5.1 to −2.3) **
Trans fatty acids (g/day)	0.8 (0.4)	−0.2 (−0.2 to −0.1) **	−0.2 (−0.3 to −0.2) **	0.9 (0.5)	−0.2 (−0.2 to −0.1) **	−0.2 (−0.2 to −0.1) **
Cholesterol (mg/day)	475.2 (144.4)	−30.7 (−48.5 to −12.8) **	−46.1 (−66.7 to −25.5) **	462.2 (139.0)	−14.6 (−33.0 to 3.8)	−15.3 (−36.0 to 5.4)
Dietary fiber (g/day)	23.8 (8.3)	1.1 (0.1 to 2.0) *	0.5 (−0.6 to 1.6)	23.0 (8.5)	0.4 (−0.5 to 1.3)	0.8 (−0.3 to 1.8)
Alcohol (g/day)	6.8 (9.0)	0.4 (−0.5 to 1.4)	0.3 (−0.8 to 1.4)	6.9 (10.2)	−0.9 (−1.8 to 0.1)	−0.6 (−1.6 to 0.3)
Energy (kcal/day)	2394.1 (676.4)	−204.2 (−285.5 to −122.9) **	−295.3 (−391.6 to −198.9) **	2360.0 (681.1)	−189.8 (−268.9 to −110.7) **	−222.9 (−310.6 to −135.1) **

* *p* < 0.05. ** *p* < 0.01.

**Table 3:** Effect of the intervention on the intake of macronutrients at 3 and 12 months.

	Mean Difference (IG–CG) 3 Months (95% CI)	Mean Difference (IG–CG) 12 Months (95% CI)	*p* for Trend
Carbohydrates (g/day)	3.8 (−11.1 to 18.7)	−4.2 (−20.8 to 12.4)	0.696
Proteins (g/day)	−0.7 (−5.1 to 3.7)	−4.8 (−9.8 to 0.2)	0.071
Total fat (g/day)	−4.0 (−9.7 to 1.8)	−4.7 (−11.7 to 2.2)	0.316
Monounsaturated fatty acids (g/day)	−2.7 (−5.7 to 0.2)	−1.9 (−5.6 to 1.7)	0.208
Polyunsaturated fatty acids (g/day)	0.5 (−0.7 to 1.8)	−0.3 (−1.9 to 1.3)	0.396
Saturated fatty acids (g/day)	−1.2 (−2.9 to 0.5)	−1.9 (−3.9 to 0.1)	0.073
Trans fatty acids (g/day)	0.0 (−0.1 to 0.0)	0.0 (−0.1 to 0.0)	0.253
Cholesterol (mg/day)	−16.1 (−41.7 to 9.5)	−30.8 (−59.9 to −1.7) *	0.043
Dietary fiber (g/day)	0.6 (−0.7 to 2.0)	−0.2 (−1.8 to 1.3)	0.431
Alcohol (g/day)	1.3 (−0.1 to 2.7)	0.9 (−0.5 to 2.4)	0.328
Energy (kcal/day)	−14.3 (−127.5 to 98.8)	−72.4 (−202.1 to 57.3)	0.470

* *p* < 0.05 compared with the baseline visit. *p*-value by ANOVA.

**Table 4:** Changes in the intake of food groups throughout the study.

	Intervention Group (*n* = 293)			Control Group (*n* =308)		
	Baseline	3 Months Mean Diff. (95 CI%)	12 Months Mean Diff. (95 CI%)	Baseline	3 Months Mean Diff. (95 CI%)	12 Months Mean Diff. (95 CI%)
Vegetables (g/day)	259.8 ± 129.7	6.9 (−7.5 to 21.4)	24.8 (7.9 to 41.7) *	255.4 ± 119.7	16.4 (3.6 to 29.3) *	25.4 (8.7 to 42.1) **
Fresh fruits (g/day)	253.4 ± 133.7	13.9 (−2.5 to 30.3)	34.3 (12.4 to 56.1) *	249.0 ± 142.3	25.3 (8.5 to 42.2) **	37.9 (16.0 to 59.8) **
Legumes (g/day)	21.2 ± 10.8	0.3 (−1.2 to 1.9)	−0.4 (−2.0 to 1.1)	20.7 ± 9.9	2.2 (0.5 to 3.9) **	0.0 (−1.9 to 1.9)
White meat (g/day)	68.4 ± 34.7	2.0 (−3.4 to 7.3)	2.2 (−3.6 to 8.0)	69.5 ± 35.8	5.9 (0.3 to 11.5) *	6.1 (−0.3 to 12.6)
Red meat (g/day)	67.0 ± 38.9	−3.6 (−8.4 to 1.1)	−9.4 (−15.9 to −2.9) **	70.6 ± 38.3	−3.1 ( −7.9 to 1.7)	−8.2 (−14.1 to −2.4) **
Fish (g/day)	107.4 ± 58.3	2.8 (−3.5 to 9.1)	−3.2 (−10.7 to 4.2)	104.8 ± 51.3	3.7 (3.7 to 11.1)	5.4 (−1.8 to 12.6)
Nuts (g/day)	12.7 ± 15.6	1.4 (−0.3 to 3.1)	2.4 (0.0 to 4.8) *	12.7 ± 16.6	−0.8 (−2.7 to 1.1)	2.4 (0.0 to 4.8)
Olive oil (g/day)	27.3 ± 18.6	−3.6 (−6.0 to −1.2) **	−2.0 (−5.0 to 1.0)	25.7 ± 17.1	−0.9 (−3.2 to 1.4)	−2.7 (−5.3 to −0.1) *
Dairy (g/day)	344.0 ± 188.5	−9.1 (−32.7 to 14.5)	−58.6 (−85.5 to −31.7) **	346.9 ± 200.3	−17.3 (−38.2 to 3.6)	−43.0 (−67.5 to −18.5) **
Full-fat dairy (g/day)	102.2 ± 107.8	−20.8 (−34.8 to −6.8) **	−26.6 (−39.8 to −13.3)	106.6 ± 119.9	−9.3 (−19.3 to 0.7)	−3.3 (−17.7 to 11.1)
Wholemeal bread (g/day)	23.4 ± 38.0	16.3 (9.7 to 22.8) **	7.2 (0.3 to 14.0) *	19.9 ± 39.3	1.5 (−3.6 to 6.7)	3.9 (−3.4 to 11.1)
Whole-grain cereals (g/day)	26.6 ± 39.5	17.4 (10.7 to 24.0) **	7.2 (−0.1 to 14.5)	22.9 ± 40.4	1.7 (−3.4 to 6.8)	3.8 (−3.4 to 11.0)
Confectionery (g/day)	48.4 ± 40.3	−21.7 (−26.8 to −16.7) **	−22.8 (−28.3 to −17.4) **	46.6 ± 40.1	−16.0 (−20.6 to −11.4) **	−17.0 (−22.5 to −11.4) **
Industrial pastries (g/day)	30.5 ± 29.5	−10.2 (−14.0 to −6.4) **	−10.2 (−14.3 to −6.2) **	31.4 ± 32.4	−8.2 (−11.6 to −4.7) **	−9.3 (−13.6 to −5.0) **
Sweetened beverages (g/day)	34.4 ± 73.0	−10.0(−19.8 to −0.1)	−12.1 (−20.6 to −3.6) **	38.2 ± 76.3	−10.2 (−21.1 to 0.7)	−15.4 (−26.5 to −4.3) **
Sodium (mg/ day)	2638.2 ± 893.0	−229.7 (−338.1 to −121.3) **	−382.6 (−503.4 to −261.8) **	2574.4 ± 894.9	−240.5 (−351.2 to −129.8) **	−268.6 (−389.2 to −147.9) **
Sugar (g/day)	8.1 ± 13.0	−1.9 (−3.5 to −0.3) *	−2.6 (−4.3 to −0.8) **	8.7 ± 14.7	−2.5 (−4.0 to −1.0) **	−2.2 (−3.8 to −0.6) **

* *p* < 0.05. ** *p* < 0.01.

**Table 5:** Effect of the intervention on the intake of food groups throughout the study.

	Mean Difference (IG–CG) 3 Months (95% CI)	Mean Difference (IG–CG) 12 Months (95% CI)	*p* for Trend
Vegetables (g/day)	−9.5 (−28.8 to 9.8)	−0.6 (−24.3 to 23.1)	0.716
Fresh fruits (g/day)	−11.5 (−34.9 to 12.0)	−3.6 (−34.5 to 27.3)	0.603
Legumes (g/day)	−1.9 (−4.2 to 0.4)	−0.4 (−2.9 to 2.0)	0.373
White meat (g/day)	−4.0 (−11.7 to 3.7)	−3.9 (−12.6 to 4.7)	0.508
Red meat (g/day)	−0.5 (−7.2 to 6.2)	−1.2 (−9.9 to 7.5)	0.528
Fish (g/day)	−0.9 (−10.6 to 8.8)	−8.6 (−18.9 to 1.7)	0.195
Nuts (g/day)	2.3 (−0.3 to 4.8)	0.1 (−3.3 to 3.4)	0.263
Dairy (g/day)	8.3 (−23.2 to 39.7)	−15.6 (−51.8 to 20.6)	0.428
Full-fat dairy (g/day)	−11.5 (−28.7 to 5.7)	−23.3 (−42.8 to −3.8) *	0.043
Olive oil (g/day)	−2.7 (−6.0 to 0.6)	0.7 (−3.3 to 4.7)	0.218
Wholemeal bread (g/day)	14.7 (6.4 to 23.0) *	3.3 (−6.7 to 13.3)	0.005
Whole-grain cereals (g/day)	15.7 (7.3 to 24.1) *	3.4 (−6.8 to 13.7)	0.004
Confectionery (g/day)	−5.7 (−12.6 to 1.1)	−5.9 (−13.7 to 1.9)	0.112
Industrial pastries (g/day)	−2.1 (−7.2 to 3.1)	−1.0 (−6.8 to 4.9)	0.304
Sweetened beverages (g/day)	0.2 (−14.4 to 14.9)	3.3 (−10.7 to 17.3)	0.860
Sodium (mg/ day)	10.8 (−143.7 to 165.3)	−114.0 (−284.3 to 56.2)	0.463
Sugar (g/day)	0.6 (−1.6 to 2.7)	−0.4 (−2.8 to 2.0)	0.293

* *p* < 0.05 compared with the baseline visit. *p*-value by ANOVA.

## Data Availability

The datasets used and/or analyzed during the current study are available from the corresponding author on reasonable request.

## Appendix A

**Table A1:** Baseline characteristics comparison between participants who completed the study and those who dropped out.

		Complete (*n* = 443)	Loss of Follow-Up (*n* = 207)	*p* Value
		Mean ± SD	Mean ± SD	
Age, years (SD)		49.2 ± 9.4	46.3 ± 9.9	<0.001
Sex, *n* (%)	Men	140 (31.6)	65 (31.4)	0.959
	Women	303 (68.4)	142 (68.6)	
Smoker status, *n* (%)	Non-smoker	181 (40.9)	82 (39.6)	0.403
	Current smoker	91 (20.5)	52 (25.1)	
	Former smoker	171 (38.6)	73 (35.3)	
Civil status, *n* (%)	Single	94 (21.2)	40 (19.3)	0.111
	Married	299 (67.5)	145 (70.1)	
	Separated	39 (8.8)	22 (10.6)	
	Widower	11 (2.5)	0 (0.0)	
BMI (kg/m^2^)		32.7 ± 3.4	33.6 ± 3.6	0.003
SBP (mmHg)		119.6 ± 15.9	119.9 ± 14.7	0.851
DBP (mmHg)		80.4 ± 9.8	79.5 ± 9.7	0.265
Total Cholesterol (mg/dL)		200.0 ± 39.1	199.4 ± 34.7	0.835
HDL Cholesterol (mg/dL)		51.6 ± 12.6	51.3 ± 12.0	0.732
Hypertension, *n* (%)		144 (32.5)	60 (29.1)	0.414
Dyslipidaemia, *n* (%)		122 (27.5)	38 (19.3)	0.029
Diabetes Mellitus, *n* (%)		5 (1.2)	4 (2.2)	0.465

BMI: Body Mass Index; SBP: Systolic Blood Pressure; DBP: Diastolic Blood Pressure.
